# High-Resolution CT Change over Time in Patients with Idiopathic Pulmonary Fibrosis on Antifibrotic Treatment

**DOI:** 10.3390/jcm8091469

**Published:** 2019-09-15

**Authors:** Elisabetta Balestro, Elisabetta Cocconcelli, Chiara Giraudo, Roberta Polverosi, Davide Biondini, Donato Lacedonia, Erica Bazzan, Linda Mazzai, Giulia Rizzon, Sara Lococo, Graziella Turato, Mariaenrica Tinè, Manuel G. Cosio, Marina Saetta, Paolo Spagnolo

**Affiliations:** 1 Department of Cardiac, Thoracic, Vascular Sciences and Public Health, University of Padova and Padova City Hospital, 35128 Padova, Italy; Elisabetta.balestro@aopd.veneto.it (E.B.); ecocconcelli@icloud.com (E.C.); dav.biondini@gmail.com (D.B.); erica.bazzan@unipd.it (E.B.); saralococo.sl@gmail.com (S.L.); graziella.turato@unipd.it (G.T.); mariaenrica.tine@gmail.com (M.T.); Manuel.cosio@mcgill.ca (M.G.C.); marina.saetta@unipd.it (M.S.); 2 Institute of Radiology, Department of Medicine, University of Padova, 35128 Padova, Italy; chiara.giraudo@unipd.it (C.G.); lindoz@gmail.com (L.M.); giulia.rizzon@hotmail.it (G.R.); 3 Istituto Diagnostico Antoniano-Affidea, 35100 Padova, Italy; rpolve@libero.it; 4 Department of Medical and Surgical Sciences, University of Foggia, Policlinico “OO. Riuniti”, 71122 Foggia, Italy; donatolacedonia@gmail.com; 5 Meakins Christie Laboratories, Respiratory Division, McGill University, Montreal, QC 65591, Canada

**Keywords:** idiopathic pulmonary fibrosis, high-resolution CT, disease monitoring, disease progression, antifibrotic treatment, therapy

## Abstract

Antifibrotic treatment slows down functional decline and disease progression in idiopathic pulmonary fibrosis (IPF). High-resolution computed tomography (HRCT) is useful to diagnose IPF; however, little is known about whether and to what extent HRCT changes reflect functional changes during antifibrotic therapy. The aim of this study was, therefore, to assess HRCT change over time after 1 year of treatment and to evaluate whether these changes correlate with functional decline over the same period of time. Sixty-eight IPF patients on antifibrotic treatment (i.e., pirfenidone or nintedanib) were functionally categorized as stable or progressors based on whether (or not) they had a decline in forced vital capacity (FVC) >5% predicted/year, and their HRCT were scored blindly and independently by two expert thoracic radiologists at treatment initiation (HRCT1) and after 1 year of treatment (HRCT2). Ground glass opacities (Alveolar Score, AS), reticulations (Interstitial Score, IS) and honeycombing (HC) were quantified and correlated with FVC decline between HRCT1 and HRCT2. At treatment initiation, HRCT scores were similar in both stable patients and progressors. After one year of treatment, in the entire population, AS and HC increased significantly, while IS did not. However, when stratified by the rate of functional decline, in stable patients, HC increased significantly while AS and IS did not. On the other hand, among progressors AS and HC increased significantly whereas IS did not. In the entire population, the combined score of fibrosis (IS + HC) correlated significantly with FVC decline. In conclusion, IPF patients on antifibrotic treatment exhibit different patterns of HRCT change over time based on their rate of functional decline. HRCT data should be integrated to lung function data when assessing response to antifibrotic treatment in patients with IPF.

## 1. Introduction

Idiopathic pulmonary fibrosis (IPF) is a chronic progressive interstitial lung disease (ILD) of unknown etiology that leads to respiratory failure and death within 3–5 years from diagnosis if untreated [1]. In addition, the clinical course of IPF patients is highly heterogeneous and largely unpredictable, with the majority of individuals experiencing a slow but inexorable decline and a minority succumbing to an acute worsening [2,3]. The 2015 ATS/ERS/JRS/ALAT guidelines conditionally recommend nintedanib and pirfenidone for the treatment of patients with IPF owing to their ability to slow down functional decline and disease progression with an acceptable safety and tolerability profile [4]. However, neither drug is a real cure for IPF, and neither drug is able to stabilize the disease or reverse fibrosis [4]. Assessment of disease severity over time and prediction of disease behavior are critically important for optimal patient management. Historically, lung function tests have been used for monitoring IPF and forced vital capacity (FVC) decline is widely accepted as a surrogate of disease progression, and possibly mortality, in IPF [5,6]. High-Resolution Computed Tomography (HRCT) is an essential component of the diagnostic work-up of IPF [3]. Indeed, the identification of a usual interstitial pneumonia (UIP) pattern on HRCT, along with the exclusion of known causes of ILD, allows a confident diagnosis of IPF to be made thus avoiding the need for histological confirmation and invasive procedures [3]. In addition, disease extent on HRCT (i.e., extent of reticular and honeycombing change) correlates with disease severity and prognosis in untreated patients with IPF [7,8,9]. However, what represents an ideal radiological scoring method to evaluate disease extension and predict progression remains highly controversial. Several semiquantitative methods of CT scoring based on visual assessment have been developed, some of which may help predict prognosis [10,11]. Yet, a number of methodological issues remain to be addressed, including the time interval needed to detect clinically meaningful changes, the correlation between radiological and functional changes and the variable (and often suboptimal) level of agreement between observers on the presence and extent of disease patterns. Inter-observer variation for the visual estimation of the extent of disease pattern is unavoidable but can be mitigated with a continuous learning method to reach a consensus [12,13]. With this background, we aimed to evaluate whether and to what extent HRCT abnormalities—as assessed by semiquantitative visual score—change after 1 year of antifibrotic treatment and how these changes correlate with different functional disease trajectories (i.e., stable patients vs. progressors) in patients with IPF.

## 2. Methods

### 2.1. Study Population and Study Design

In this restrospective longitudinal study, we analyzed a cohort of phenotypically well characterized patients with IPF referred to our center between April 2014 and April 2018 and followed clinically, functionally (FVC, forced vital capacity in one second, FEV_1_, forced expiratory volume in one second and diffusing capacity of the lung for carbon monoxide (DL_CO_)) and radiologically for at least one year after initiation of anti-fibrotic treatment (either pirfenidone or nintedanib).

Sixty-eight patients were included from two ILD centers in Italy (University Hospital of Padova, *n* = 59 and University Hospital of Foggia, *n* = 9). For all patients, the diagnosis of IPF was made in accordance with the ATS/ERS/JRS/ALAT guidelines [2,3]. Thirty-three cases required a histological confirmation of the diagnosis of IPF, whereas, in the remaining cases (*n* = 35), the diagnosis was made based on clinical and radiological data only. Patients with a clear history of environmental or occupational exposure and those with clinical features or serological data suggestive of an underlying connective tissue disease were excluded.

For all patients, clinical and lung function data were collected at the time of treatment initiation and at regular time intervals (every three months) for up to 12 months while HRCT was performed at treatment initiation and after 12 months (Table 1). Based on their annual rate of decline in absolute FVC% pred., patients were classified as progressors (absolute FVC% pred. decline/year > 5%, *n* = 20) or stable (absolute FVC% pred. decline/year ≤ 5%, *n* = 48). Improvement of FVC (%pred. and mL) was expressed as a negative value.

The study was performed in accordance with the Declaration of Helsinki and was approved by the Ethics Committee of the University Hospital of Padova (4280/AO/17). Informed consent was obtained for all study participants.

### 2.2. Radiological and Functional Analysis

For each patient, an HRCT was available at treatment (either pirfenidone or nintedanib) initiation (HRCT1) and at the 12-month follow-up (HRCT2). The HRCTs were performed by a 64 slice Siemens Somatom Sensation (Siemens Healthcare, Erlangen, Germany), applying a slice thickness ≤1.5 mm.

Two expert thoracic radiologists, who were blind to clinical and functional data and timing of HRCT, scored HRCT1 and HRCT2 images independently using a semi-quantitative scale. This represented a modification of the previously reported scoring systems [14,15] that allowed to evaluate “reticulation” more precisely. Specifically, the radiologic features considered in this study were ground glass opacities (GGO) (alveolar score, AS), reticulation (interstitial score, IS) and honeycombing (HC) (honeycombing score, HC). For each lung lobe, the two radiologists assessed the extent of AS, IS and HC using a scale from 0–100 and estimated the extent to the nearest 5%. After each individual lobe was scored, the result was expressed as the mean value of the five lobes in AS, IS and HC. Finally, the IS and HC were pooled (IS + HC) to analyze the amount of fibrotic abnormalities. The level of interobserver agreement was obtained for each patient as a mean of 5 lobes and for each radiologic abnormality (i.e., IS, AS and HC) and expressed as Cohen’s k value. Disagreement between radiologists was resolved by consensus. The correlation between radiological change and FVC decline was calculated as the change in AS (ΔAS/month), IS (ΔIS/month), HC (ΔHC/month), pooled IS and HC (ΔIS + HC/month) and the change in FVC milliliters (mL) per month (ΔFVC mL/month) and FVC% pred. per month (ΔFVC% pred./month) between HRCT1 and HRCT2 [15].

### 2.3. Statistical Analysis

Categorical variables are described as absolute (n) and relative values (%), whereas continuous variables are described as median and range. To compare demographic data and baseline clinical characteristics between stable patients and progressors, a Chi square test and Fisher’s exact test for categorical variables and a Mann–Whitney U test for continuous variables were used as appropriate.

Wilcoxon signed rank test was performed to compare HRCT1 and HRCT2 for the grading scores of different variables (AS, IS, HC and IS + HC) in the entire population, in stable patients and progressors. Correlation coefficients between radiological and functional data were calculated using the nonparametric Spearman’s rank method. The level of interobserver agreement between the two radiologists was evaluated by kappa statistic measure [16].

The overall survival was calculated from diagnosis to death or lung transplantation with data censured at 1 June 2019. The cumulative survival rate was calculated using a Kaplan–Meier method and clinical characteristics and radiological scores were evaluated to determine their relationship with disease progression in a univariate and multivariate analysis of Cox proportional hazards regression testing (Supplementary Materials).

All data were analyzed using SPSS Software version 25.0 (New York, NY, USA: IBM Corp. USA). *p*-values < 0.05 were considered statistically significant.

## 3. Results

### 3.1. Clinical, Functional and Radiological Evaluation at Baseline

**Figure 1:**
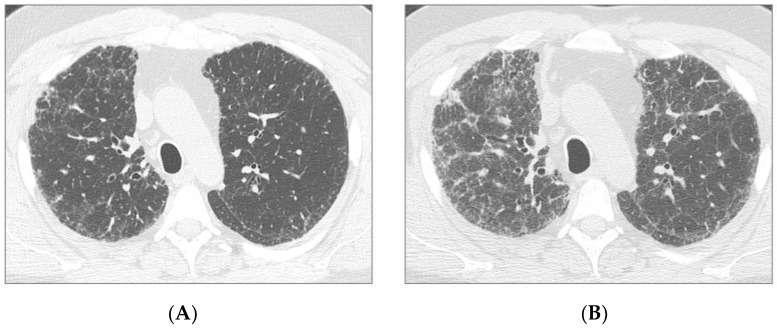

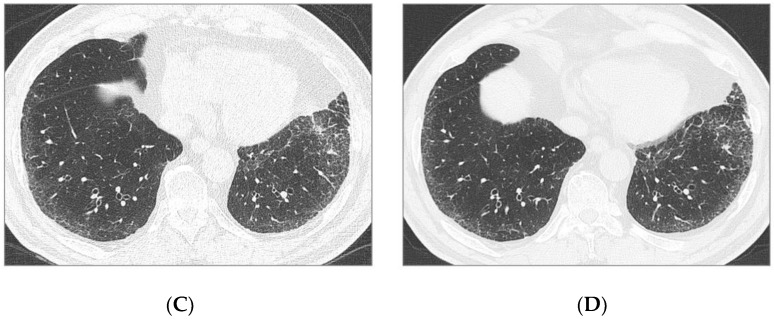
Axial HRCT images of two patients: a 53 year-old male with a progression of disease (patient 1) (**a**,**b**) and a 63 year-old male with stable disease (patient 2) (**c**,**d**). Patient 1: HRCT at treatment start (**a**) and after one year of treatment (**b**), demonstrating a significant progression of ground glass opacities and reticulation. Patient 2: HRCT at treatment start (**c**) and after one year of treatment (**d**), demonstrating stability of ground glass opacities and reticulation.

Sixty-eight patients with IPF were included in the study (Table 1). Most patients were males (81%) and former smokers (59%) with a median age at diagnosis of 66 years (range 44–78). Based on the annual FVC% pred. decline during treatment over the study period, 48 patients were classified as stable (stable FVC or FVC% pred. decline/year ≤ 5%) and 20 as progressors (FVC% pred. decline/year > 5%) (Table 1, Figure 1).

At treatment initiation, sex, smoking history and % of radiological diagnosis did not differ between stable patients and progressors, while progressors tended to be younger and with significantly more preserved FVC and DL_CO_ as compared to stable patients. At treatment initiation, there were no between-group differences in the HRCT score (Table 1). Forty-seven patients were treated with pirfenidone and twenty-one with nintedanib and none of them discontinued treatment due to adverse effects during the study period.

### 3.2. Functional and Radiological evaluation

**Figure 2:**
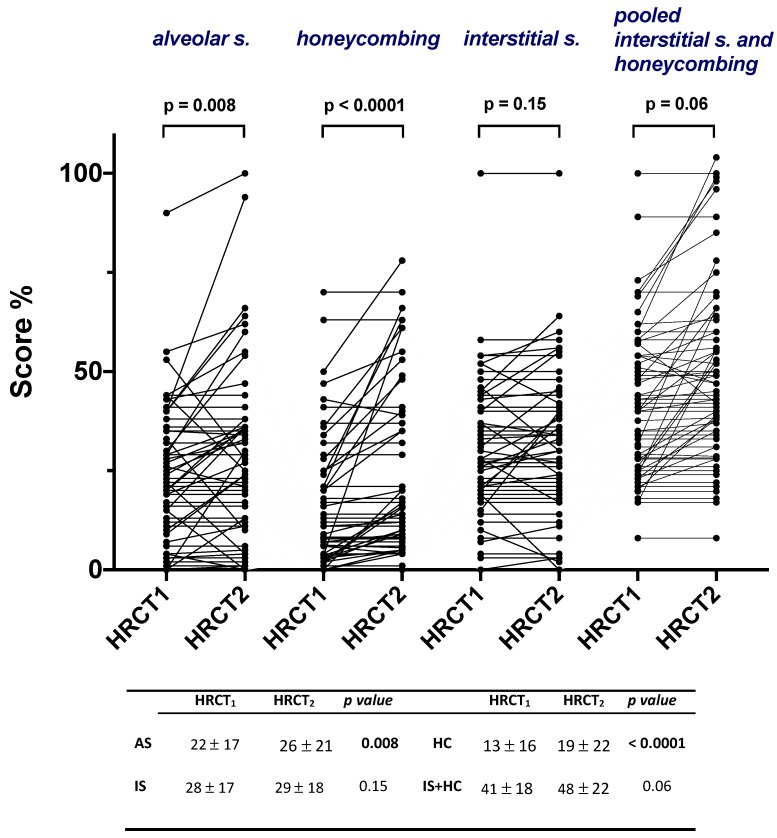
Alveolar score, interstitial score, honeycombing and pooled interstitial score and honeycombing at treatment initiation (HRCT1) and after one year of treatment (HRCT2) in the entire study population. Values in the table below are expressed as mean and standard deviations. P values refer to comparisons between HRCT1 and HRCT2.

Overall, the inter-observer agreement between the two radiologists with regard to change in AS, IS and HC was good (Cohen’s kappa = 0.71 for IS, k = 0.76 for AS, k = 0.80 for HC). In the entire study population, AS and HC increased significantly between HRCT1 and HRCT2 from 22% ± 17% to 26% ± 21% (*p* = 0.008) and from 13% ± 16% to 19% ± 22% (*p* < 0.0001), respectively (Figure 2).

**Figure 3:**
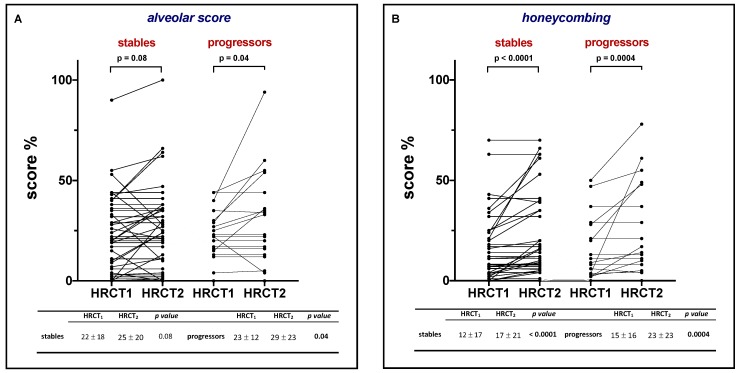

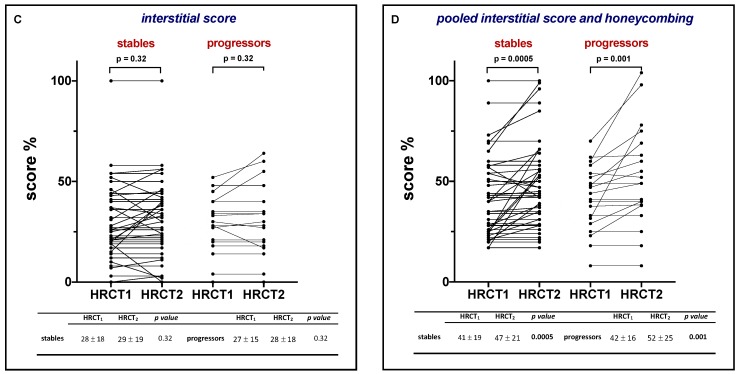
Change in alveolar score, honeycombing, interstitial score and pooled interstitial score and honeycombing between HRCT1 (at treatment initiation) and HRCT2 (after one year of treatment) in *stable* patients (*n* = 48) and progressors (*n* = 20). Values in the table below are expressed as mean and standard deviations. P values refer to comparisons between HRCT1 and HRCT2.

When the study population was stratified by the rate of functional decline, in stable patients, HC increased significantly between HRCT1 and HRCT2 from 12% ± 17% to 17% ± 21% (*p* < 0.0001) (Figure 3, Panel B), whereas AS and IS did not (Figure 3, Panel A–C). Conversely, among progressors, both AS and HC increased significantly from 23% ± 12% to 29% ± 23% (*p* = 0.04) and from 15% ± 16% to 23% ± 23% (*p* = 0.0004), respectively (Figure 3, Panel A–B), whereas IS did not (Figure 3, Panel C).

When IS and HC were pooled together, the IS + HC score increased significantly both in stable patients (from 41% ± 17% to 47% ± 21%, *p* = 0.0005) and progressors (from 42% ± 16% to 52% ± 25%, *p* = 0.001), respectively (Figure 3, Panel D). Finally, when radiologic scores were analyzed for the nintedanib and pirfenidone group separately, after 1 year of treatment, AS increased significantly in the pirfenidone but not in the nintedanib group (*p* = 0.013 and *p* = 0.36, respectively). On the other hand, HC increased significantly in both the nintedanib and pirfenidone group (0.007 and *p* < 0.0001, respectively), whereas IS did not

### 3.3. Functional and Radiological Correlations

**Figure 4:**
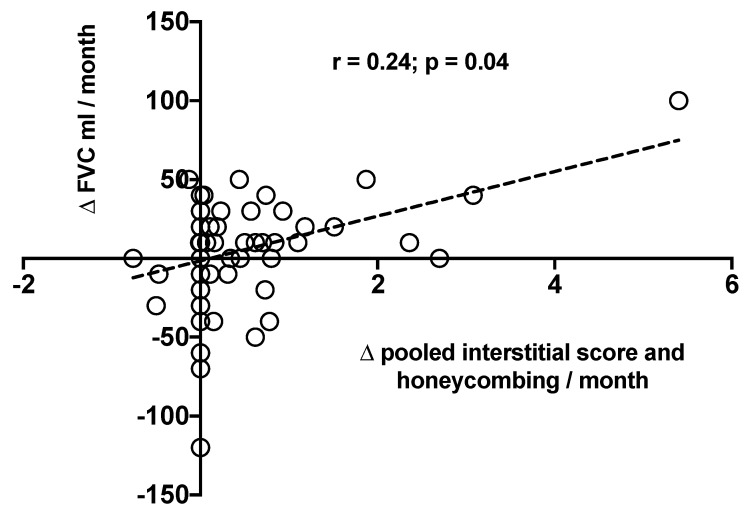
Correlation between change over time in FVC mL (ΔFVC mL/month) and change over time in the pooled Interstitial Score and Honeycombing (Δ pooled Interstitial Score and Honeycombing) in the entire study population. Negative values mean improvement of FVC.

In the entire study population, we observed a positive correlation between ΔFVC mL/month and ΔIS + HC/month (r = 0.24, *p* = 0.04) (Figure 4), while none of the correlations between ΔFVC mL/month and ΔAS, ΔIS and ΔHC were significant (r = 0.10, *p* = 0.40; r = −0.04, *p* = 0.60 and r = −0.07, *p* = 0.50, respectively). When stratified by FVC decline (stable patients vs. progressors), the correlation between ΔFVC mL/month and ΔIS + HC/month was not confirmed in either group (r = 0.14, *p* = 0.32; r = 0.40, *p* = 0.07, respectively).

The previously observed correlation between ΔFVC and ΔIS + HC/month was confirmed when the change in FVC was expressed as ΔFVC% predicted (r = 0.25, *p* = 0.04), whereas the correlations between ΔFVC% pred./month and ΔAS, ΔIS and ΔHC were not significant (r = 0.01, *p* = 0.90; r = 0.19, *p* = 0.15 and r = 0.04, *p* = 0.7, respectively). Similarly, there was no significant correlation between ΔFVC% pred./month and ΔIS + HC/month, neither in patients functionally stable nor in progressors (r = 0.40, *p* = 0.07 and r = 0.15, *p* = 0.29, respectively).

For survival analysis, univariate and multivariate analysis of Cox proportional hazards regression testing, see Supplementary Materials.

## 4. Discussion

The present study aimed to assess whether and to what extent radiologic abnormalities evolve after 1 year of antifibrotic treatment and whether these changes correlate with different trajectories of disease course—as assessed by lung function—in patients with IPF stratified by their rate of functional decline (stable vs. progressors). In our study population, antifibrotic therapy slowed down the rate of FVC decline; indeed, IPF patients under treatment lost, on average, approximately 100 mL/year of FVC, which mirrors what has been observed both in clinical trials [17,18] and in a number of real-world studies [19,20,21]. However, despite this clear treatment effect on lung function after 1 year of treatment, we also observed an increased extension of HRCT abnormalities both in terms of alveolar opacity and honeycombing (Figure 2). In addition, the observed correlation between the combined score of fibrosis (IS + HC) and FVC decline is in keeping with previous studies [22,23] and supports the concept that patients with IPF experience an inexorable disease progression—both functional and radiological.

IPF patients display a heterogeneous (and unpredictable) disease course, namely slow or rapid progression [24,25,26]. In a cohort of IPF patients stratified in slow and rapid progressors based on their pretreatment rate of FVC decay, we have recently shown that the beneficial effect of antifibrotic treatment (pirfenidone) differed significantly between the two phenotypes, being significantly more pronounced in the rapidly progressive group [27]. Given this clear between-group difference in treatment response, in this study, we investigated whether and to what extent the assessment and quantification of HRCT patterns of disease may identify disease progression, including different responses to antifibrotic treatment. Similar to previous studies, we stratified our IPF patients under treatment in stable and progressors based on an FVC loss ≤ and > 5% [21,28] and analyzed longitudinally the type and extent of HRCT changes in these two patient subgroups. At the start of treatment, HRCT scores were similar in both stable and progressors. Among progressors, both AS and HC increased significantly after 1 year of treatment, whereas IS did not (Figure 3). On the other hand, in stable patients, HC increased significantly, while AS and IS did not (Figure 3). The observation that HC tends to progress in both stable patients and progressors confirms previous findings in untreated patients and demonstrates that the overall extent of lung fibrosis on CT (combination of reticulation and honeycombing) is a proxy of disease severity, as well as representing a strong independent predictor of mortality in patients with IPF [23]. Notably, the extent of honeycombing at baseline and its progression over time are also important determinants of mortality in patients with fibrosing ILD other than IPF [29].

Our study shows that progressors displayed a significant increase of AS over time despite treatment, whereas stable patients did not. This is an interesting finding, although the significance of alveolar opacity or ground glass attenuation remains debated. The term “ground-glass attenuation” refers to the presence of a hazy and diffuse homogeneous increase in lung density and, when located akin dense fibrotic areas, may represent mild/initial fibrosis [30]. However, ground glass attenuation may also be associated with the presence of inflammatory cells in the alveolar or interstitial space (i.e., alveolitis) [31,32,33], which is often more evident in cases with more aggressive disease. In support of this possibility is our recent observation that the different clinical course (rapid or slow) of untreated IPF patients undergoing lung transplantation is associated with distinct underlying pathology in the explanted lungs [26]. In particular, as compared to slow progressors, rapid progressors showed an extensive cellular immune/inflammatory infiltrate [26]. Moreover, we have also demonstrated that the alveolar score on HRCT may reflect the extent of the alveolar infiltrate as suggested by its correlation with the total number of lymphocytes in the explanted lungs [15]. Notably, untreated patients experiencing a rapid functional decline have, at baseline, a higher alveolar score than slow progressors [15]. This finding coupled with the observation that rapid progressors despite treatment also exhibit an increased extension in alveolar score suggests that the alveolar score may help to identify, even early in the disease course, the more aggressive IPF phenotype and supports the routine use of CT and its visual characterization in clinical practice both in treated and untreated patients with IPF. When we analyzed radiologic scores in the nintedanib and pirfenidone groups separately, we found that after 1 year of treatment, AS increased significantly in the pirfenidone (*p* = 0.013) but not in the nintedanib group (*p* = 0.36). Whether this difference is real or is simply due to the smaller number of patients in the nintedanib group (n = 21 vs. 46 in the pirfenidone group) is difficult to ascertain. Answering this question would require a larger dataset that, currently, is not available.

Currently, longitudinal HRCT is used predominantly in clinical practice to identify complications of IPF, such as lung cancer or indirect signs of pulmonary hypertension. To the best of our knowledge, this is the first study that explores the role of change over time in CT scores and its correlation with functional decline in IPF patients on antifibrotic therapy. Our findings, we believe, are of particular interest as they may potentially help in early detection of disease progression by identifying subtle abnormalities that are not captured by a lung function test.

Only Iwasawa et al. have investigated longitudinal radiologic abnormalities during treatment in patients with IPF [34]. The authors reported the utility of quantitative CT analysis for predicting the efficacy of pirfenidone. They compared treated and untreated IPF patients and found that the change in fibrotic lesions was significantly smaller among pirfenidone-treated patients compared to controls and that the decline in vital capacity (VC) correlated significantly with the increase in fibrotic lesions [34].

The findings of our study should be interpreted in light of some limitations, such as the relatively small number of patients. Nevertheless, our study population was larger than that evaluated in previous studies of IPF patients on antifibrotic treatment and, of note, our patients were followed-up longitudinally with a serial lung function test and HRCT, which were scored by two expert thoracic radiologists with good interobserver agreement. Secondly, we did not perform automated quantitative imaging analysis [35] as this tool is not available at our institution. However, the good agreement of the readers demonstrates that the proposed score is robust and guarantees reliable results. Furthermore, visual analysis continues to play a key role in diagnosing, monitoring and assessing disease severity in IPF [13]. In this regard, Robbie and colleagues have recently reviewed pros and cons of automated and manual CT measurements of lung volume [36] and concluded that lung volume (i.e., volume loss) and extent of fibrosis on CT correlate significantly with pulmonary function test parameters of lung volume irrespective of whether visual or automated techniques are used, and may, therefore, be complementary measures for disease monitoring in IPF [36]. Moreover, as elegantly pointed out by Wu X and colleagues, these software analyses have themselves, in any case, some limitations and disadvantages such as the applicability to retrospective CT datasets [37]. Finally, the follow-up time was relatively short (i.e., 12 months), and we are currently in the process of collecting functional and radiological data over a longer period of time.

In conclusion, in patients with IPF on antifibrotic treatment, the extent of honeycombing increases over time both in patients experiencing functional decline and in those who remain functionally stable over 12 months, suggesting that CT is able to capture subtle subclinical disease progression. Longitudinal HRCT evaluation may, therefore, provide important information that integrate those provided by lung function and clinical evaluation.

## Figures and Tables

**Table 1:** Patients demographics and clinical characteristics.

	Entire	Stables	Progressors	*p-*Value
	Population			
	(*n* = 68)	(*n* = 48)	(*n* = 20)	
Male—*n* (%)	55 (81)	37 (77)	18 (90)	0.31
Female—*n* (%)	13 (19)	11 (23)	2 (10)	0.31
Age at diagnosis—*years*	66 (44–78)	68 (46–78)	61 (44–78)	0.07
Smoking history—*pack years*	15 (0–80)	15 (0–80)	15 (0–55)	0.31
Current—*n* (%)	9 (13)	7 (15)	2 (10)	1.00
Former—*n* (%)	40 (59)	29 (60)	11 (55)	1.00
Nonsmokers—*n* (%)	19 (28)	12 (25)	7 (35)	0.55
Clinical-radiological diagnosis—*n* (%)	35 (51)	27 (56)	8 (40)	0.29
Histological diagnosis—*n* (%)	33 (49)	21 (44)	12 (60)	0.29
FVC at diagnosis—*L*	2.76 (1.19–5.68)	2.6 (1.19–5.29)	2.97 (1.68–5.68)	0.04
FVC at diagnosis—% *pred.*	78 (44–120)	78 (44–120)	78 (50–107)	0.40
FEV1 at diagnosis—*L*	2.21 (1.02-4.45)	2.19 (1.02–4.45)	2.50 (1.40–3.70)	0.06
FEV1 at diagnosis—% *pred*.	83 (40–127)	83 (40–127)	86 (49–122)	0.27
DL_CO_ at diagnosis—% *pred.*	57 (34–114)	53 (34–114)	65 (37–97)	0.02
6MWT at diagnosis—mt	400 (125–600)	400 (125–600)	408 (250–540)	0.50
FVC decline per year—*mL*	86 (−1381–1155)	37 (−1381–371)	413 (135–1155)	<0.0001
FVC decline per year—% *pred.*	2 (−25–29)	0 (−25–4.7)	9 (5–29)	<0.0001
Deaths—*n* (%)	16 (23)	8 (17)	8 (40)	0.05
Alveolar score in HRCT1—*%*	21 (0–90)	21 (0–90)	22 (0–44)	0.68
Honeycombing in HRCT1—*%*	7 (0–70)	6 (0–70)	9 (0–50)	0.32
Interstitial score in HRCT1—*%*	26 (0–100)	26 (0–100)	28 (0–52)	0.92
Pooled interstitial score and honeycombing—*%*	40 (8–100)	38 (17–100)	43 (8–70)	0.52

Values are expressed as numbers and (%) or median and ranges as appropriate. Negative values mean improvement of FVC (Forced Vital Capacity). To compare demographic data and baseline clinical characteristics between stable and progressors, Chi square test and Fisher t test (*n* < 5) for categorical variables and Mann–Whitney t test for continuous variables were used.

## Supplementary Materials

The following are available online at https://www.mdpi.com/2077-0383/8/9/1469/s1, Figure S1: Survival analysis of stables and progressor patients, Table S1: Predictive factors of overall survival in the entire population of IPF patients treated with antifibrotics.

## Abbreviations

IPF	Idiopathic Pulmonary Fibrosis
UIP	Usual Interstitial Pneumonia
HRCT	High-Resolution Computed Tomography
GGO	Ground Glass Opacities
FVC	Forced Vital Capacity
DLCO	diffusing capacity of the lung for carbon monoxide
6MWT	six-minute walking test
L	liters
mL	milliliters
FEV_1_	forced expiratory volume in one second
IS	Interstitial Score
AS	Alveolar Score
HC	honeycombing
