# Supplementary material for: High-Resolution CT Change over Time in Patients with Idiopathic Pulmonary Fibrosis on Antifibrotic Treatment
Source: J Clin Med. 2019 Sep 15;8(9):1469. doi: 10.3390/jcm8091469 (PMC6780456; doi:10.3390/jcm8091469)
Supplement: Supplementary file 1 [file jcm-08-01469-s001.pdf]

# Supplementary Material

*Article*

# High-Resolution CT Change over Time in Patients with Idiopathic Pulmonary Fibrosis on Antifibrotic Treatment

**Elisabetta Balestro [1,†], Elisabetta Cocconcelli [1,†], Chiara Giraudo [2], Roberta Polverosi [3], Davide Biondini [1], Donato Lacedonia [4], Erica Bazzan [1], Linda Mazzai [2], Giulia Rizzon [2], Sara Lococo [1], Graziella Turato [1], Mariaenrica Tinè [1], Manuel G. Cosio [1,5], Marina Saetta [1] and Paolo Spagnolo [1,*]**

1 Department of Cardiac, Thoracic, Vascular Sciences and Public Health, University of Padova and Padova City Hospital, 35128 Padova, Italy; Elisabetta.balestro@aopd.veneto.it (E.B.); ecocconcelli@icloud.com (E.C.); dav.biondini@gmail.com (D.B.); erica.bazzan@unipd.it (E.B.); saralococo.sl@gmail.com (S.L.); graziella.turato@unipd.it (G.T.); mariaenrica.tine@gmail.com (M.T.); Manuel.cosio@mcgill.ca (M.G.C.); marina.saetta@unipd.it (M.S.)
2 Institute of Radiology, Department of Medicine, University of Padova, 35128 Padova, Italy; chiara.giraudo@unipd.it (C.G.); lindoz@gmail.com (L.M.); giulia.rizzon@hotmail.it (G.R.)
3 Istituto Diagnostico Antoniano-Affidea, 35100 Padova, Italy; rpolve@libero.it
4 Department of Medical and Surgical Sciences, University of Foggia, Policlinico "OO. Riuniti", 71122 Foggia, Italy; donatolacedonia@gmail.com
5 Meakins Christie Laboratories, Respiratory Division, McGill University, Montreal, 65591 QC, Canada
* Correspondence: paolo.spagnolo@unipd.it
† These two authors contributed equally to this work as first authors.

Received: 17 August 2019; Accepted: 11 September 2019; Published: date

## Methods

*Statistical analysis*

The overall survival was calculated from diagnosis to death or lung transplantation with data censured at June 1st, 2019. The cumulative survival rate was calculated using Kaplan-Meier method and the difference in the survival time between the two groups (stable and progressors) was assessed with log-rank test. Clinical characteristics and radiological scores were evaluated to determine their relationship with disease progression in a univariate analysis of Cox proportional hazards regression testing. Variables with an association statistically significant or almost significant ($0.05 < p < 0.09$) with overall survival at univariate analysis were included in a multivariate Cox proportional hazard regression test to find the factors independently associated with disease progression.

## Results

*Survival analysis and association between clinical – radiological parameters and survival*

Survival of stable patients was not statistically different from survival of progressors (HR 1.93, 95% CI 0.85 - 4.41; p= 0.11) (**Figure S1**).

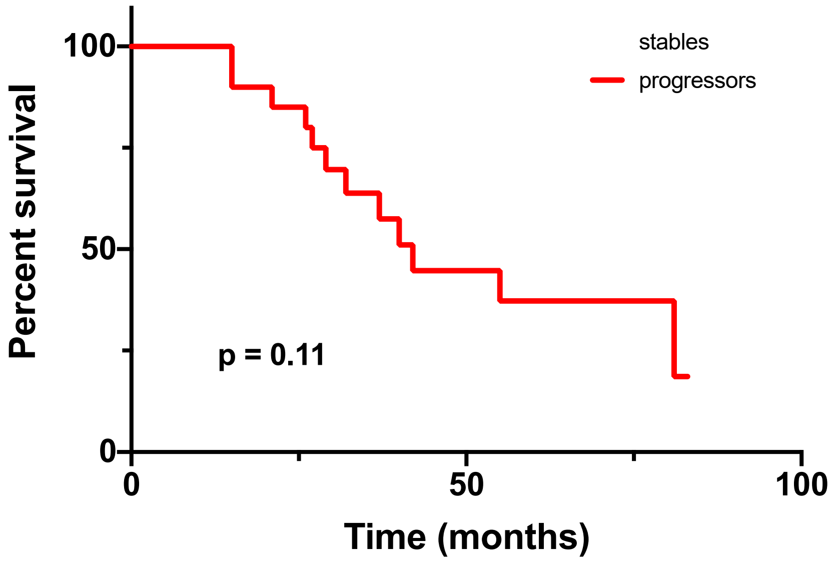


**Figure S1.** Survival analysis of stables and progressor patients. The gray line represents the survival in the stables and the red line represents the survival in the progressors. Kaplan Meier analysis was used with a log-rank test (HR 1.93, 95% CI 0.85 - 4.41; p=0.11).

To detect factors predictive of disease progression in the entire IPF population, we used Cox proportional hazards regression analysis. Univariate analysis of factors associated with survival revealed that FVC (liters (L)) at diagnosis, FEV1 (L) at diagnosis, DLCO after one year of antifibrotic drug, FVC (L) and FVC % pred. after one year of antifibrotic drug, 6-minute walking test (6MWT) after one year of antifibrotic drug and IS+HC in HRCT1 had significant positive association with disease progression in the entire IPF population (**Table S1**). Of interest, univariate analysis of factors associated with survival showed that 6MWT at diagnosis, 6MWT change over one year of treatment, ΔHC, IS+HC in HRCT2 had an almost significant positive association with disease progression. Multivariate analysis performed using variables having statistical significance or almost significant in univariate analysis, revealed that only 6MWT at diagnosis (HR: 3.64; 95%CI: 1.16 – 11.42; p = 0.03) and 6MWT change over one year of treatment (HR: 0.32; 95%CI: 0.11 – 0.91; p = 0.03) are independent predictors of disease progression in IPF patients.

**Table S1**. Predictive factors of overall survival in the entire population of IPF patients treated with antifibrotics

| | **Univariate analysis** | | **Multivariate analysis** | |
|---|---|---|---|---|
| | **HR (95% CI)** | ***p* Value** | **HR (95% CI)** | ***p* Value** |
| **Disease progression (*stables* vs. *progressors*)** | 0.55 (0.26 – 1.17) | 0.12 | - | - |
| **Sex (*male* vs. *female*)** | 0.90 (0.36 – 2.24) | 0.82 | - | - |
| **Age at diagnosis (*years ≥66* vs. *< 66*)** | 1.02 (0.48 – 2.20) | 0.94 | - | - |
| **Smoking history (*pack years≥15* vs. <15)** | 1.67 (0.75 – 3.70) | 0.20 | - | - |
| **Smoking status (*no* vs *current* vs. *former*)** | 1.40 (0.90 – 2.18) | 0.13 | - | - |
| **FVC at diagnosis (≥ *2.76 L* vs. *< 2.76*)** | 0.34 (0.15 – 0.76) | **0.009** | 2.63 (0.63 – 10.87) | 0.18 |
| **FVC at diagnosis (≥*78%* vs. < 78%)** | 0.66 (0.31 – 1.40) | 0.28 | - | - |
| **FEV$_1$ at diagnosis (≥*83%* vs. *< 83%*)** | 0.7 (0.33 – 1.47) | 0.34 | - | - |
| **FEV$_1$ at diagnosis (≥*2.21 L* vs. *< 2.21 L*)** | 0.43 (0.20 – 0.95) | **0.037** | 0.58 (0.13 – 2.51) | 0.46 |
| **DL$_{CO}$ at diagnosis (≥*57%* vs. *< 57%*)** | 0.84 (0.40 – 1.76) | 0.64 | - | - |
| **DL$_{CO}$ after 1-yr of antifibrotic drug (≥*48%* vs. *< 48%*)** | 0.40 (0.18 – 0.90) | **0.03** | 1.01 (0.31 – 3.27) | 0.98 |
| **DL$_{CO}$ change (Δ) (≥*4.5%* vs. *< 4.5%*)** | 1.36 (0.64 – 2.90) | 0.42 | - | - |
| **FVC after 1-yr of antifibrotic drug (≥*75%* vs. < 75%)** | 2.28 (1.03 – 5.06) | **0.04** | 0.85 (0.25 – 2.86) | 0.80 |
| **FVC after 1-yr of antifibrotic drug (≥*2.6L* vs. *< 2.6L*)** | 2.66 (1.17 – 6.07) | **0.02** | 1.83 (0.52 – 6.39) | 0.34 |
| **FVC decline after 1-yr of antifibrotic drug (≥*86ml* vs. *< 86ml*)** | 1.03 (0.45 – 2.37) | 0.93 | - | - |
| **6MWT at diagnosis (≥*400 mt* vs. *< 400 mt*)** | 0.51 (0.23 – 1.11) | 0.09 | 3.64 (1.16 – 11.42) | **0.03** |
| **6MWT after 1-yr of antifibrotic drug (≥*400 mt* vs. *< 400 mt*)** | 0.40 (0.18 – 0.88) | **0.02** | 0.81 (0.26 – 2.55) | 0.72 |
| **6MWT change (Δ) (≥*20 mt* vs. *< 20 mt*)** | 2.24 (0.97 – 5.17) | 0.05 | 0.32 (0.11 – 0.91) | **0.03** |
| **Alveolar score in HRCT1 (≥*21%* vs *< 21%*)** | 1.54 (0.72 – 3.29) | 0.26 | - | - |

| | | | | |
|---|---|---|---|---|
| **Alveolar score in HRCT2 (≥*22%* vs < *22%*)** | 1.17 (0.55 – 2.48) | 0.68 | - | - |
| **Alveolar score change (Δ) (> *0%* vs ≤*0%*)** | 1.28 (0.60 – 2.71) | 0.51 | - | - |
| **Honeycombing in HRCT1 (≥*7%* vs < *7%*)** | 0.96 (0.45 – 2.03) | 0.91 | - | - |
| **Honeycombing in HRCT2 (≥*7%* vs < *7%*)** | 1.13 (0.53 – 2.39) | 0.75 | - | - |
| **Honeycombing change (Δ) (> *0%* vs ≤*0%*)** | 2.10 (0.99 – 4.46) | 0.05 | 0.52 (0.22 – 1.23) | 0.14 |
| **Interstitial score in HRCT1 (≥*26%* vs < *26%*)** | 1.73 (0.79 – 3.74) | 0.16 | - | - |
| **Interstitial score in HRCT2 (≥*27%* vs < *27%*)** | 1.29 (0.60 – 2.76) | 0.51 | - | - |
| **Interstitial score change (Δ) (> *0%* vs ≤*0%*)** | 0.61 (0.24 – 1.52) | 0.29 | - | - |
| **Interstitial s. and honeycombing in HRCT1 (≥*26%* vs < *26%*)** | 0.27 (0.10 – 0.67) | **0.005** | 0.32 (0.08- 1.16) | 0.08 |
| **Interstitial s. and honeycombing in HRCT2 (≥*26%* vs < *26%*)** | 0.47 (0.21 – 1.04) | 0.06 | 1.39 (0.46 – 4.22) | 0.55 |
| **Interstitial s. and honeycombing change (Δ) (> *0%* vs ≤*0%*)** | 0.80 (0.37 – 1.69) | 0.56 | - | - |

Values are expressed as HR (95%CI). Univariate and multivariate Cox proportional hazard regression tests were used to determine the relationship of clinical, functional and radiological characteristics with disease progression.
